# European survey on national training activities in clinical research

**DOI:** 10.1186/s13063-019-3702-z

**Published:** 2019-10-29

**Authors:** A. Magnin, V. Cabral Iversen, G. Calvo, B. Čečetková, O. Dale, R. Demlova, Gy. Blasko, F. Keane, G. L. Kovacs, C. Levy-Marchal, E. C. Monteiro, L. Palmisano, D. Pella, A. Portolés Pérez, O. Rascol, C. Schmid, F. Tay, H. von der Leyen, C. Ohmann

**Affiliations:** 1 SCTO - Swiss Clinical Trial Organisation, Bern, Switzerland; 2 NorCRIN – Norwegian Clinical Research Infrastructures Network, Trondheim, Norway; 3 SCReN – Spanish Clinical Research Network, Madrid, Spain; 4 SLOVACRIN – Slovak Clinical Research Infrastructure Network, Bratislava, Slovakia; 5 CZECRIN – Czech Clinical Research Infrastructure Network, Prague, Czech Republic; 6 HECRIN – Hungarian Clinical Research Infrastructure Network, Budapest, Hungary; 7 HRB CRCI – Health Research board, Clinical Research Coordination Ireland, Dublin, Ireland; 8 F-CRIN – French Clinical Research Infrastructure Network, Paris, France; 9 PTCRIN - Portuguese Clinical Research Infrastructure Network, Lisboa, Portugal; 10 ItaCRIN - Italian Clinical Research Infrastructure Network, Rome, Italy; 11 KKSN – Netzwerk der Koordinierungszentren für Klinische Studien, Hannover, Germany; 12 ECRIN – European Clinical Research Infrastructure Network, Kaiserswerther Str, 70 40477 Düsseldorf, Germany

**Keywords:** Clinical research, Clinical study, Clinical trial, Survey, Training, GCP-training, ECRIN, Career options, Training requirements, Clinical study personnel

## Abstract

**Background:**

Investigator-initiated clinical studies (IITs) are crucial to generate reliable evidence that answers questions of day-to-day clinical practice. Many challenges make IITs a complex endeavour, for example, IITs often need to be multinational in order to recruit a sufficient number of patients. Recent studies highlighted that well-trained study personnel are a major factor to conduct such complex IITs successfully. As of today, however, no overview of the European training activities, requirements and career options for clinical study personnel exists.

**Methods:**

To fill this knowledge gap, a survey was performed in all 11 member and observer countries of the European Clinical Research Infrastructure Network (ECRIN), using a standardised questionnaire. Three rounds of data collection were performed to maximize completeness and comparability of the received answers. The survey aimed to describe the landscape of academic training opportunities, to facilitate the exchange of expertise and experience among countries and to identify new fields of action.

**Results:**

The survey found that training for Good Clinical Practice (GCP) and investigator training is offered in all but one country. A specific training for study nurses or study coordinators is also either provided or planned in ten out of eleven countries. A majority of countries train in monitoring and clinical pharmacovigilance and offer specific training for principal investigators but only few countries also train operators of clinical research organisations (CRO) or provide training for methodology and quality management systems (QMS). Minimal requirements for study-specific functions cover GCP in ten countries. Only three countries issued no requirements or recommendations regarding the continuous training of study personnel. Yet, only four countries developed a national strategy for training in clinical research and the career options for clinical researchers are still limited in the majority of countries.

**Conclusions:**

There is a substantial and impressive investment in training and education of clinical research in the individual ECRIN countries. But so far, a systematic approach for (top-down) strategic and overarching considerations and cross-network exchange is missing. Exchange of available curricula and sets of core competencies between countries could be a starting point for improving the situation.

## Background

Investigator-initiated clinical studies (IITs) help to answer questions that clinicians face in their day-to-day practice. They are initiated and managed in a non-commercial setting by a researcher/s (individual investigator, academic institution, a collaborative study group, etc). IITs play a vital role in the generation of evidence that can eventually drive policy. A good IIT is one that asks a question that has scientific merit, has robust research methodology, has rigorous ethical and scientific governance, a committed and motivated investigator, institutional support, a funding agency that recognizes the value of the research and a team that backs him/her up [1]. However, there are many challenges posed by an IIT, as has recently been shown with the example of Portugal. Besides limited funds and missing local infrastructure, a lack of trained study personnel is seen as a major hurdle [2, 3]. More and more, IITs need to be multinational (e.g. in order to recruit sufficient numbers of patients), which increases their complexity.

Many important publications have emphasised and confirmed the importance of training and education to improve the quality, impact and efficiency of clinical research in general. The Lancet series on “Research: increasing value, reducing waste” [4–8] mentions that training and education are important measures for increased impact of clinical research results on medical therapies. The OECD says that support for and facilitation of the education, training and infrastructure required for academic clinical trials are key elements of the success of clinical research [9]. Qualified and experienced staff are essential at several levels for conducting clinical trials, as the German Scientific Council states [10].

But what does the European landscape for training in clinical research look like? The National Scientific Partners of the European Clinical Research Infrastructure Network (ECRIN) are a major provider of training in clinical research and well informed on activities in their own countries. Therefore, a survey in ECRIN member and observer countries was performed to map the status of training activities at the national level in the National Scientific Networks. However, considerable heterogeneity between the partners with respect to involvement in national training activities has been reported. ECRIN is a sustainable, non-profit, distributed infrastructure with the legal status of a European Research Infrastructure Consortium (ERIC). The vision of ECRIN is to generate scientific evidence with a focus on IITs to optimise medical standards of care. To this end, ECRIN supports investigators and (mainly) academic sponsors in the planning and design of multinational clinical research [11]. ECRIN has nine member countries and two observer countries (Table 1).

**Table 1:** National Clinical Research Networks belonging to ECRIN

Country	ECRIN national scientific partner
Czech Republic	CZECRIN—Czech Clinical Research Infrastructure Network
France	F-CRIN—French Clinical Research Infrastructure Network
Germany	KKSN—Network of Coordination Centres for Clinical Studies
Hungary	HECRIN—Hungarian Clinical Research Infrastructure Network
Ireland	HRB CRCI—Health Research Board, Clinical Research Coordination Ireland
Italy	ItaCRIN—Italian Clinical Research Infrastructure Network
Norway	NorCRIN—Norwegian Clinical Research Infrastructure
Portugal	PtCRIN—Portuguese Clinical Research Infrastructure Network
Slovakia*	SLOVACRIN—Slovak Clinical Research Infrastructure Network
Spain	SCReN—Spanish Clinical Research Network
Switzerland*	SCTO – Swiss Clinical Trial Organisation

*Observer country

Scientific partners of ECRIN are national clinical research networks that are composed of academic clinical research centres (CRCs) or clinical trial units (CTUs) with a national coordinating centre (Table 1) [12]. As per ECRIN-ERIC statutes, the scientific partners must have developed shared tools, procedures and practices to facilitate multi-centre studies, have reached a critical mass in competence and represent the standard in the country [13].

Objectives of the survey:

- To describe the landscape of academic training opportunities in clinical research as described by the ECRIN partners
- To enable synergies by facilitating the exchange of expertise and experience in national training in clinical research
- To identify new fields of action for ECRIN to increase the quality and efficiency of multinational clinical research

In Additional file 1 a longer description of ECRIN is included.

## Methods

The survey was initiated in April 2018. A first version of the questionnaire was sent to the ECRIN members on 20 April 2018. The answers were compiled and presented at the NC meeting on 14 May 2018. It was decided to provide a first internal report and to give the individual countries the opportunity to go back to the questions and complete/update if needed.

As a consequence, the interim report and an updated questionnaire with a more standardised answer format were distributed to the members of the NC on 15 June 2018 with a deadline of 13 July 2018. On 14 August 2018, a reminder was sent to countries which had not yet provided the filled-in questionnaire. On 4 October 2018 the second round of data collection was completed and the next version of the report was finalised on 19 October 2018. The information provided was considered being of high value, but still too heterogenous to be shared with a broader audience. Therefore, the NC agreed to perform a third revision of the report, which was completed in January 2019.

## Results

### Overview

All members/observers from ECRIN participated in the survey. Table 2 gives an overview of the answers received.

**Table 2:** Overview of the training activities for clinical research in each country

Training activity	Country										
	CZE	FRA	DEU	HUN	IRL	ITA	NOR	PRT	SVK	ESP	CHE
GCP	X	X	X	X	X		X	X	X	X	X
Study nurse/coordinator	X		X	X	X	X	(X)	X	(X)	X	X
Investigator	X	X	X	X	X	X		X	X	X	X
Monitoring	X	X	X	X			X	X		X	X
PV/clinical pharmacology	X		X	X				X	(X)	X	X
Principal investigator	X	X	X	X				X	X		X
CRO operators			X			X		X			
Methodology		X			X			X		X	X
QMS	X	X	X		X			X			
Postgraduate	X		X	X	X		X	X	X		X
Other		X	X	X	X						X

X = available, (X) = planned

*CZE* Czech Republic, *FRA* France, *DEU* Germany, *HUN* Hungary, *IRL* Ireland, *ITA* Italy, *NOR* Norway, *PRT* Portugal, *SVK* Slovakia, *ESP* Spain, *CHE* Switzerland, *GCP* Good Clinical Practice, *PV* pharmacovigilance, *CRO* clinical research organization, *QMS* quality management system

#### GCP training

Specific (basic and advanced) GCP training is provided by ECRIN members/observers in all but one country. The training is usually targeted at investigators, study nurses/coordinators and members of the research team and normally provided by the local CTU. In Germany it is provided as an introductory or refresher course. Standardised curricula are applied in Czech Republic (network standard), Hungary (accredited course, covered partly by the Hungarian Society of Pharmacology, with exams, which is valid for 5 years), Portugal (CLIC PharmaTrain [14]/ECRIN [15]), Slovakia and Switzerland (accredited by swissethics). In Ireland GCP training is targeted at IMP and medical devices (mandatory). GCP courses for investigators are mandatory in Germany, Hungary, Ireland, Norway, Portugal and Switzerland and are not mandatory in France and Spain, though GCP must be well known.

In some countries, there is a collaboration with commercial clinical research organisations (CROs) for GCP training (e.g. Norway, Portugal), some of them with Transcelerate recognition [16].

#### Study nurse/coordinator training

Specific study nurse, respectively coordinator training is provided or planned in ten countries (in preparation in Slovakia and Norway). In Czech Republic, Germany, Hungary, Portugal, Spain and Ireland this training is optional; in Italy it is mandatory. The training is provided by CTUs (Germany, Ireland and Portugal), private companies (Italy and Portugal) or a mixed consortia (Hungary). Only in Germany is a standardised curriculum applied. Norway and Switzerland are in the planning phase for a curriculum.

#### Investigator training

Ten countries offer investigator training. Mandatory investigator courses are performed by local CTUs in Germany, Ireland and Switzerland and by a consortium in Hungary. A standardised curriculum has been implemented in Germany by KKSN and Portugal (CLIC level 1 plus level 2 for principal investigators (PIs) [14]). In Czech Republic, the training is optional.

#### Monitoring training

Monitoring training based upon a standardised curriculum is used in Czech Republic (mandatory for CZECRIN CRAs, provided by CZECRIN or an external agency) and Germany (optional, provided by CTUs). Monitoring training is also offered in France, Hungary, Norway, Portugal (by PTCRIN CTUs or external organizations), Spain and Switzerland (eight countries in total).

#### Pharmacovigilance training/training in clinical pharmacology

Training in clinical pharmacology is implemented in Germany, Hungary, Portugal and Spain. In Hungary it is intended for PIs and mandatory for phase 1 trials and optional for phase 2–3 trials. It is based upon standardised curricula and allows certification. The MD degree in clinical pharmacology should be regularly enforced with exams every 5 years. In CZECRIN, mandatory pharmacovigilance (PV) courses are provided by an external agency for CZECRIN staff based upon a standardised curriculum. Norway has had a medical speciality of clinical pharmacology taking 5 years since 1989. Portugal has a medical specialization with 4 years of training. Slovakia is planning a PV course for investigators and study nurses. In Switzerland, a MD can acquire a recognised specialty title in Clinical Pharmacology and Toxicology, duration 5 years.

#### PI/coordinator training

Seven countries perform PI/ coordinator training. The PI training offered in Germany and Portugal is optional, based on a standardised curriculum by KKSN or PTCRIN/UNAVE (Aveiro University [14]) and provided by the CTUs (Germany) or by e-learning platform (Portugal). In addition, there is optional and non-standardised training for study coordinators. In Switzerland, mandatory PI/sponsor-investigator training is available.

#### Training of CRO operators (staff operating or working for a CRO)

Italy has comprehensive, mandatory training for CRO operators for all activities, including monitoring, quality, GCP, good manufacturing practice (GMP) and PV (mainly for phase I trials). Germany and Portugal offer training for CRO operators as well.

#### Methodological training

In Ireland, a wide range of training in trial methodology based upon national curricula is provided and performed nationally through HRB TMRN. Also, Spain, Portugal (CLIC level 2 [14] and others) and Switzerland offer such training. France has a specific methodological training in Parkinson’s disease. In a collaboration between Harvard Medical School and the Portuguese government, Portugal has implemented a clinical scholars research training programme dedicated to medical doctors [17].

#### Quality management system training

Quality management system (QMS) training is provided in Czech Republic, France (optional), Portugal (optional), Germany and Ireland (mandatory and provided nationally through HRB-CRCI).

#### Postgraduate training

There are several activities with respect to postgraduate training. In Czech Republic, a mandatory clinical trials course for PhD students, using a standardised PhD curriculum, is provided by local CZECRIN staff and senior clinical researchers. Germany has a broad spectrum of master programmes, including a Master of Clinical Trial Management (MSc), a “Clinical Evaluation” study programme, a master of science in “Clinical Research & Translational Medicine”, a master of science in “Medical Biometry/Biostatistics” and a master of science in “Pharmaceutical Medicine”. Ireland [18–22] and Switzerland [23] also provide different optional postgraduate training courses in clinical research (MSc courses and other postgraduate courses, including a specialty title in Pharmaceutical Medicine for MDs and dedicated PhD programmes for clinical researchers). Portugal has implemented a master in clinical research management (MEGIC) [24] and has several PhD and master courses on epidemiology and clinical research. The latter is offered in Norway as well. Postgraduate courses in Hungary and Slovakia were not further specified. Table 3 provides complementary information.

**Table 3:** Career options and academic titles in clinical research

Country	Career options and academic titles for clinical research
Czech Republic	Career options: study nurses, coordinators, researchers, biostatistician, methodologist Academic titles: none
France	Career options: positions with academic sponsors and CTUs, but poor progression in careers, low salaries, insufficient recognition. Options: clinical research associate (CRA), project manager, data manager Academic titles: none General remarks: poor and not specific, no specific training for MDs, technical personnel; no recognition of clinical research know-how for hospital career
Germany	Career options: **e**mployment in academic research institutions, pharmaceutical industry, authorities or CROs Academic titles: [25–32] MS Clinical Research & Translational Medicine, Universität Leipzig (part time) MS Clinical Trial Management, Beuth Hochschule für Technik, Berlin (part time) MS Pharmaceutical Medicine, Universität Duisburg-Essen (part time) MS Medical Biometry/Biostatistics, Universitäts Heidelberg (part time) MS Clinical Research, International University Dresden (part time) MS Clinical Research, Centrial Tübingen, berufsbegleitend MBA Clinical Research Management, Internationale Hochschule (study at a distance) BSc Medical Research and Management, Medical School Berlin (full time)
Hungary	All four medical schools offer various PhD training programmes in clinical research. Our aim is that the CTUs could select well-trained staff from the students who have achieved outstanding results during participation in the HRDOP training courses. The goal is also to increase the number and the quality of human clinical trials by developing and implementing a co-constructed training curriculum, from students participating in medical training to the clinical study team
Ireland	Career options: careers in clinical research in Ireland are generally funded through programmatic funding or individual study funding, rather than through core permanent funding, which limits the career pathways for those involved in clinical research. With the development of the national infrastructure, i.e. Clinical Research Facilities, respectively. centres and national thematic networks, more secure roles and career paths have developed but the majority of this infrastructure is also funded through programmatic funding (generally 3-year terms) rather than core permanent funding, which is problematic in terms of career paths. Career options correspond to those offered in Spain and there are further options in CRO’s, pharma and medical device companies Academic titles: see [18–22].
Italy	Career options at CROs, private companies, hospital/academic clinical trial centres, no academic titles in clinical research
Norway	No specific career options but, based on acquired competence, positions in research departments/CTUs are available. In some hospitals part-time functions as research leaders are available at the department level Academic titles: none
Portugal	Career options: study nurse/study coordinator, clinical research assistant/CRA, project manager, auditor/quality manager, data manager, bio-statistician, clinical investigator Academic titles: PhD in Clinical Research and Health services (Faculdade de Medicina, Univ. Porto) PhD in Experimental and Clinical Pharmacology and Toxicology (Faculdade de Medicina, Univ. Porto) PhD in Medicine (six Universities in Portugal) Medical competency in “Pharmaceutical Medicine” (Portuguese Medical Association) Medical specialist in “Clinical Pharmacology” (Ministry of health) MSc (Master of Science) in: • Clinical Research Management (Nova Medical School, Univ. Nova Lisboa) • Medicines and Health Products: Regulatory and Evaluation (Faculdade de Farmácia, Univ. Lisboa) • Applied Pharmacology (Faculdade de Farmácia, Univ Coimbra) • Epidemiology (Faculdade de Medicina, Univ. Lisboa) • Biostatistics and Bioinformatics applied to health (Escola Superior de Tecnologias da Saúde, Porto)
Slovakia*	Career options: clinical study coordinator in hospitals Academic titles: none
Spain	Career options: study nurse, clinical investigator, study coordinator, data entry, CT pharmacist, CTA, CRA, project manager, clinical trial manager, data manager, bio-statistician, pharmacovigilance, methodologist, quality management Academic titles: Biostatistics (University), Clinical Pharmacology—medical specialty (Health Ministry), different private or university specific courses on monitoring and/or project management
Switzerland* (see also [23])	Career options: study nurse/study coordinator, clinical research assistant/CRA, project manager, auditor/quality manager, data manager, bio-statistician, clinical investigator Academic titles: PhD in clinical research Federal physician specialist title “Pharmaceutical Medicine” MAS (Master of advanced studies): • MAS in Drug Discovery and Clinical Development • Master in Medicines Development DAS (Diploma of Advanced Studies): • Diploma in Pharmaceutical Medicine [33] • DAS Clinical Trial Practice and Management • DAS (Diploma of advanced studies) Clinical Research • DAS (Diploma of advanced studies) Management of Clinical Trials CAS (Certificate of Advanced Studies): • CAS Clinical Research I–Clinical Trial Planning and Conduct • CAS Clinical Research II (Advanced Clinical Trial Management) • CAS Clinical Research Coordinator • CAS patient oriented Clinical Research • CAS in Clinical Data Management • CAS in Clinical Monitoring • CAS in Clinical Trial Management

*Observer

#### Other types of training, general remarks

France offers MD training courses focused on good practice and technical issues (optional). Ireland offers two optional training programmes aimed at medical device development, training and education. In Germany, an advanced training course for clinical studies with medical devices is offered (complementary to the investigator training and following a standardised curriculum).

Other specific training activities were reported from several countries. Germany offers training courses in medical English for study personnel as well as for people handling dangerous goods. Hungary offers project-specific training, HRDOP [34], ERASMUS [35]. France offers a broad range of specific training courses in e.g. imaging, European trials, study drug issues, basic information for patients (responsible representatives), risk management, rare diseases, human sample collection, and data management [36]. Furthermore, a “train the trainer” program is offered for clinical research [37].

In Spain, besides GCP and methodology, all training is internal for SCReN members (and mandatory). In Switzerland, a comprehensive list showing all clinical research training provided by the network is available (see also [23]).

### Minimal requirements for a study specific function (e.g. CRA, biostatistician)

Minimal requirements for study-specific functions cover GCP in ten countries: Czech Republic (PI, study team members), France, Germany (study personnel), Hungary, Ireland (clinical research staff), Norway (PI), Portugal (clinical research staff), Slovakia (investigators), Spain (PI) and Switzerland. Additional requirements have been reported for phase I units in France and Hungary [38], for CRO operators in Italy and for PIs and sponsor PIs in Switzerland.

### National requirements for continuous education in clinical research

Eight countries have national requirements for continuous training. Repeated GCP training is required in Czech Republic, Germany, Ireland and Spain (commonly every 2 years), in Portugal (every 3 years), Hungary (every 5 years) and Slovakia. Swissethics recommends that after the initial Training in Research Ethics and GCP has taken place, the knowledge is maintained by regular research activity, e.g. conducting clinical trials, attending continuing education related to research or GCP refresher courses, being exposed to GCP inspections, etc. In Hungary, participation in yearly educational programmes is highly advised and in an ongoing project the qualification requirements and the duration of their validity will be determined for the clinical trial team in human clinical studies. In Germany, refresher courses every 2 years for medical devices are mandatory. In Spain, in phase 1 trial units, certification for cardiovascular resuscitation and emergency treatment is required. No national requirements were reported from France, Italy and Norway.

### Overarching national strategy/roadmap/standards for training in clinical research

In four countries, an overarching national strategy is in place or foreseen for training: Germany (GCP training for investigators and study personnel according to the curriculum of the Federal Association of Physicians and ethics committees), Hungary (Committee on Clinical Pharmacology and Medical ethics of the Medical Research School), Ireland (Health Research Board strategy for development of the national network and funding of HRB-CRCI and HRB Trials Methodology Research Network (TMRN)) and Switzerland (Federal Office of Public Health (FOPH) 2016–20,121 roadmap for building up the future generation of clinical researchers encompasses a total of five work packages, the SCTO contributes to the implementation [39]). In Hungary, the training materials (curriculum, admission and exclusion criteria) resulting from the HRDOP project will be presented to the Medical Research Council (ETT) and the National Institute of Pharmacy and Nutrition (OGYÉI) as well. No national strategy has been specified for France, Italy, Norway, Portugal and Slovakia. In Czech Republic, a national strategy is prepared within the Working Committee for Clinical Trials at the Ministry of Health and for Spain no information is available.

### Career options for clinical research per country

In Table 3 career options and academic titles for clinical research are listed per country.

Career options for clinical research are still limited in the majority of countries. The same is true for academic titles, respectively degrees specific for clinical research; however, new options for masters and diplomas have been created.

### Necessity for complementary international training in clinical research

All countries participating in the survey support the idea of offering international training in clinical research. Concrete proposals were made by Czech Republic (MSc programmes in “Pharmaceutical Medicine”), France (master degree for PIs of multinational studies), Germany (specific management related to international research projects, e.g. PharmaTrain, where Hungary organised two courses in pharmaceutical development), Portugal (CLIC III for sponsor-coordinators and face to face courses focusing on key topics, e.g. protocol design, H2020), Spain (international regulatory requirement training, international clinical trials coordination, VHP and new centralised evaluation of clinical trials under the new regulation (536/2014) and Switzerland (training for “lead” CTUs to facilitate multinational trials). E-learning courses would be of interest for Czech Republic and France. A coordination role of ECRIN in this area is proposed by Germany, Hungary, Ireland and Norway. Certification would be an option for Ireland and Italy (if certification is recognised at the national level). Slovakia has no suggestions yet.

## Discussion

The European countries included all have academic training opportunities in clinical research, but to different extents. The respondents are open to foster the exchange of expertise and experience in national training in clinical research as well as for a more active role of ECRIN to identify new fields of action, increasing the quality and efficiency of multinational clinical research.

The investment of the national and local networks in training and education is substantial and impressive. But so far, building up training and education programmes is rather a bottom-up process serving current needs. Furthermore, there are few systematic approaches for (top-down) strategic and overarching considerations, active promotion and cross-network exchange. However, the EU just recently funded an ERASMUS+ project (CONSCIOUS: Curriculum Development of Human Clinical Trials for the Next Generation Biomedical Students). This consortium is led by Hungary in collaboration with France, Ireland, Czech Republic and Portugal and is intended to tackle skill gaps and mismatches related to European-level clinical trial professionals [﻿23﻿].

More exchange between National Scientific Networks of ECRIN member and observer countries may inspire their national plans, projects and negotiations. The information may furthermore serve as a benchmark to position, scrutinise and further develop their own organisation. The national view may be challenged and enriched by the broader European picture. And last but not least, access to information, documents and expertise should lead to a mutual benefit for all members, avoiding duplication of efforts and a waste of taxpayer money. Showing and promoting the impact and (untapped) potential of a network of networks may help to support the continuation of national funding. ECRIN and the scientific partners may also support the European Union (respectively the responsible taskforces) in continuously harmonising training once the Clinical Trial Regulation (EU number 536/2014) enters into force.

Curricula for clinical research functions/determination of core competencies for specific functions (e.g. for universal functions like (principal) investigator or study nurse) and a European-wide agreement on curricula and core competencies could be an aim. As a starting point, the exchange of available curricula and sets of core competencies could improve professionalism.

Training offered by ECRIN should be distinguished between “internal training” (for the CTUs and networks) and “external training” (e.g. for clients like investigators). Whereas the latter may preferably be offered at the national or even local level (may be supported by central offers for multinational, European or international aspects), internal training at the European level could be of high value.

The national networks are increasingly creating and offering jobs for highly qualified staff. Furthermore, academic career paths are starting to be offered more systematically and are officially recognised, resulting in titles (e.g. PhD in clinical research). This will lead to more professionalism in clinical research and will attract more young talents. ECRIN may map and monitor this development and facilitate staff exchanges between national networks.

There are some limitations of the survey. Data collection has been restricted to 11 member and observer countries of ECRIN and the coordinating units of the National Scientific Networks. The ECRIN partners served as a convenient and representative sample. However, we cannot exclude other training initiatives not known to the network. As a consequence, the information may not be complete in all cases and may not be representative for the European Union. In addition, heterogeneity in the use of clinical research terms was identified in the survey. As an example, different countries have a different understanding of the profile of a study coordinator. For some countries this means “coordinating investigator”, for others it means rather a project manager function or is a synonym for study nurse. Defining the different profiles in advance may help to get a more consistent result in the future. The same is true for academic titles and degrees (PhD, master, etc.) versus career options (CRA, data manager, etc.). Nevertheless, the survey has been performed in three rounds with stepwise improvement of the information and face to face discussions between the survey participants and thus gives a good overview of the training activities in the participating countries (see Additional file 2).

## Conclusions

There is a substantial and impressive investment in training and education of clinical research in the individual ECRIN countries. But so far, a systematic approach for (top-down) strategic and overarching considerations and cross-network exchange is missing. Exchange of available curricula and sets of core competencies between countries could be a starting point for improving the situation.

## Supplementary information

**Additional file 1:** Background information about ECRIN. Short overview on support areas, organisation and history of ECRIN. (PDF 233 kb)

**Additional file 2:** Good practice in the conduct and reporting of survey research. (DOCX 14 kb)

## Data Availability

All data generated or analysed during this study are included in this published article.

## Abbreviations

CLIC: Clinical investigator certificate
CR: Clinical research
CRA: Clinical research associate
CRC: Clinical research centre
CRO: Clinical research organisation
CT: Clinical trial
CTA: Clinical trial application
CTU: Clinical trial unit
DM: Data management
ECRIN: European Clinical Research Infrastructure Network
ERASMUS: EuRopean Community Action Scheme for the Mobility of University Students
ERIC: European Research Infrastructure Consortium
GCP: Good Clinical Practice
GDPR: General Data Protection Rule
GMP: Good Manufacturing Practice
H2020: Horizon 2020
ICN: International Clinical Trial Network
IIT: Investigator-initiated trial (or study)
IMP: Investigational medicinal product
MD: Medical doctor
MSc: Master of science
NC: Network committee
OECD: Organisation for Economic Co-operation and Development
PhD: Doctor of philosophy
PI: Principal investigator
PV: Pharmacovigilance
QA: Quality assurance
QM: Quality management
QMS: Quality management system
SAS: Self-assessment sheet
SOP: Standard operating procedure
WG: Working group
