# Supplementary material for: European survey on national training activities in clinical research
Source: Trials. 2019 Oct 29;20:616. doi: 10.1186/s13063-019-3702-z (PMC6821032; doi:10.1186/s13063-019-3702-z)
Supplement: Supplementary file 1 — Additional file 1: Background information about ECRIN. Short overview on support areas, organisation and history of ECRIN. (PDF 233 kb) [file 13063_2019_3702_MOESM1_ESM.pdf]

European survey on national training activities in clinical research

Additional file 1: Background information about ECRIN

Overview

The European Clinical Research Infrastructure Network (ECRIN, www.ecrin.org) is a sustainable, non-profit, distributed infrastructure with the legal status of a European Research Infrastructure Consortium (ERIC).

ECRIN supports multinational clinical trials in Europe, which provide increased access to patients, resources and expertise. Multinational trials generate potentially more robust trial results and may have greater public health impact. Moreover, multi-site and multinational collaboration reduce bias and improve generalisability.

Support areas: clinical trials, tools, data centre certification, infrastructure development (projects)

ECRIN offers diverse study support services, which are mainly provided to multinational trials initiated by investigators and biotech and medical device small and medium enterprises (SMEs). In particular, ECRIN supports investigators and sponsors for trial planning (e.g., advice on funding applications and trial design, from site selection to logistics and insurance issues), the validation of study protocols (scientific review, feasibility and risk assessment), and trial management (regulatory and ethical authorisation, adverse event reporting, monitoring, data management, etc.).

As part of its trial-support activities, ECRIN develops and maintains freely accessible tools such as databases on regulatory and ethical issues, outcome measures, and risk-based monitoring. Such activities are intended to enhance the ability of European sponsors to successfully conduct multi-country clinical research. Another activity that aims to achieve this purpose is data centre certification, an ECRIN quality programme that provides independent certification to data centres across Europe and beyond. The programme identifies non-commercial CTUs that have demonstrated that they can provide safe, secure, compliant and efficient management of clinical research data. It does so by auditing the units for compliance with published ECRIN data standards. ECRIN-certified centres are recommended for data management in ECRIN-supported trials.

Moreover, ECRIN is involved in infrastructure development projects that aim to further develop the European clinical research community, facilitate multinational trials, and foster international cooperation in non-commercial trials.

Organisation

ECRIN’s organisational model is based on country membership. It currently has nine member countries (Czech Republic, France, Germany, Hungary, Ireland, Italy, Norway, Portugal and Spain) and two observer countries (Slovakia and Switzerland).

In each member and observer country, ECRIN has a National Scientific Partner: a network of academic clinical research infrastructures (i.e. academic clinical trial units, CTUs, and/or clinical research centres, CRCs), which are located in the main national universities and hospitals. The staff working in these units are highly experienced and are very familiar with the local and national research environment.

Each member and observer country hosts a European Correspondent (EuCo), a clinical research expert. Typically seconded to ECRIN by their local research institution, EuCos are familiar with the national clinical trial landscape and ensure efficient coordination of multinational trials. In particular,

they manage the clinical trial portfolio and coordinate the services provided by the National Scientific Partner, with support from the Paris-based core team. EuCos (and the core team) typically perform centralised trial management tasks, while national partners perform decentralised tasks. To ensure quality and harmony of work, all EuCos use the same tools and quality standards.

The ECRIN core team – in addition to supporting the EuCos by coordinating their work with each other and partners as needed – develops ECRIN's strategy as well as common tools and procedures for ECRIN-supported trials, and contributes to other infrastructure development projects.

History

ECRIN began in 2004 as a consortium and matured through European Union Framework Program 6 and 7 (FP6 and FP7) and H2020 funding (projects awarded in 2004, 2006, 2008, 2012 and 2017). In 2006, it was listed on the European Strategy Forum on Research Infrastructures (ESFRI) roadmap. After identification of the bottlenecks in multinational trials, ECRIN began to create common tools as well as regulatory and ethical know-how (e.g., databases) to enhance collaboration. It started to provide services for multinational clinical studies in 2009. In parallel, to support this work, it established networks with academic CTUs across Europe. This cross-border coordination enabled the development and implementation of ECRIN's fourth project, an FP7-funded project (grant agreement number 284395) called "ECRIN Integrating Activity," or ECRIN-IA. The project involved 23 countries and aimed to develop partnerships with investigator communities in three areas of high relevance for interventional clinical research: rare diseases, medical devices and nutrition.

In November 2013, ECRIN was awarded ERIC status by the European Commission. This is a legal status recognised in all EU member states, designed to facilitate the establishment and operation of research infrastructures of European interest. In 2016, ECRIN was designated as an ESFRI Landmark. Landmarks are research infrastructures (RIs) that are considered "pan-European hubs of scientific excellence, generating new ideas and pushing the boundaries of science and technology". The attribution of Landmark status is a major accomplishment for ECRIN as it reflects its valuable and long-term contribution to the European research landscape through the provision of high-quality scientific and managerial services.

In terms of other major projects, ECRIN followed up on ECRIN-IA with a new H2020 project focused on pediatrics: Pediatric Clinical Research Infrastructure Network (PedCRIN) (grant agreement number agreement number 731046), launched at the start of 2017.

Other partners

In addition to its member and observer countries, ECRIN works with many other partners across the globe. These groups or organisations are typically focused on clinical research in general, or on a specific research area or disease in particular. They may be directly involved in ECRIN-supported trials or collaborative projects, or may also simply wish to maintain close contact with ECRIN and support its goals. ECRIN's partners may be designated as affiliate or international partners, depending on the nature of the collaboration and agreement signed (i.e., framework agreement or MoU).

HOW ECRIN WORKS WITH NATIONAL PARTNERS

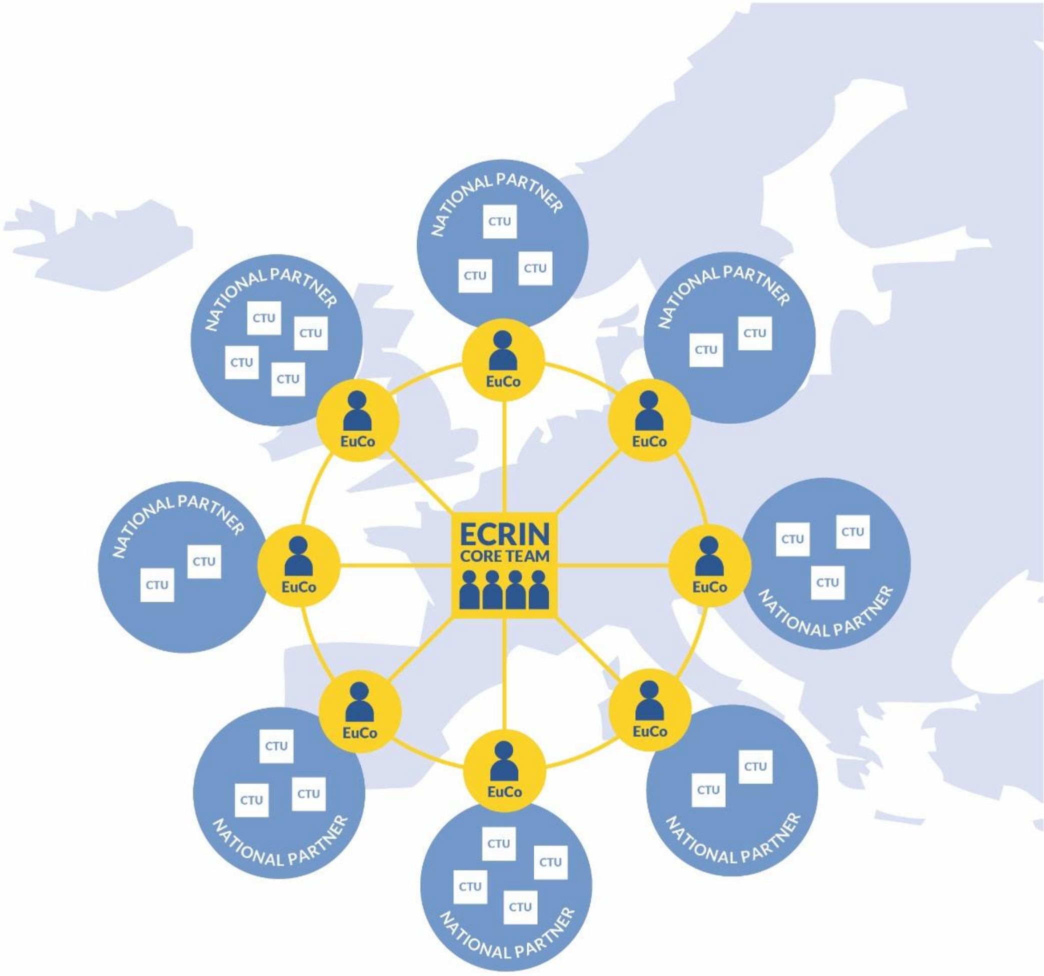

EuCo European Correspondent | **CTU** Clinical Trial Unit
