# Supplementary material for: European survey on national training activities in clinical research
Source: Trials. 2019 Oct 29;20:616. doi: 10.1186/s13063-019-3702-z (PMC6821032; doi:10.1186/s13063-019-3702-z)
Supplement: Supplementary file 2 — Additional file 2: Good practice in the conduct and reporting of survey research. (DOCX 14 kb) [file 13063_2019_3702_MOESM2_ESM.docx]

**Good practice in the conduct and reporting of survey research**

(Kelley K, Clark B, Brown V, Sitzia J. Good practice in the conduct and reporting of survey research. Int J Qual Health Care. 2003;15(3):261-266)

| Reporting Item |  |  |  |  |
| --- | --- | --- | --- | --- |
| **Background** |  |  |  |  |
| Justification of research method | Yes |  |  |  |
| Background literature review | Yes |  |  |  |
| Explicit research question | Yes |  |  |  |
| **Methods** |  |  |  |  |
| Description of methods used for data analysis | Yes |  |  |  |
| Location of data collection | Yes |  |  |  |
| Number and types of contact | Yes |  |  |  |
| Representativeness | Yes |  |  |  |
| Method of sample selection | Yes |  |  |  |
| **Research tool** |  |  |  |  |
| Description of the research tool | Yes |  |  |  |
| Description - development of research tool | Yes |  |  |  |
| Instrument pretesting | Yes |  |  |  |
| Instrument reliability and validity | Yes |  |  |  |
| **Results** |  |  |  |  |
| Results of research presented | Yes |  |  |  |
| Results address objectives | Yes |  |  |  |
| **Response rates** |  |  |  |  |
| Response rate stated | Yes (100%) |  |  |  |
| **Interpretation and discussion** |  |  |  |  |
| Interpret and discuss findings | Yes |  |  |  |
| Conclusions and recommendations | Yes |  |  |  |
| Limitations | Yes |  |  |  |
| **Ethics and disclosure** |  |  |  |  |
| Consent | Yes |  |  |  |
